# Early upper digestive tract side effects of zidovudine with tenofovir plus emtricitabine in West African adults with high CD4 counts

**DOI:** 10.7448/IAS.16.1.18059

**Published:** 2013-04-30

**Authors:** Eric Ouattara, Christine Danel, Raoul Moh, Delphine Gabillard, Gilles Peytavin, Romuald Konan, Jérome Le Carrou, Franck Bohoussou, Serge P Eholie, Xavier Anglaret

**Affiliations:** 1 Programme PACCI, Abidjan, Côte d'Ivoire; 2 Univ. Bordeaux, ISPED, F-33000 Bordeaux, France; INSERM, Centre INSERM U897, F-33000 Bordeaux, France; 3 Service de Pharmacologie, Hôpital Bichat Claude-Bernard, Paris, France; 4 Service des Maladies Infectieuses et Tropicales, CHU de Treichville, Abidjan, Côte d'Ivoire

**Keywords:** sub-Saharan Africa, antiretroviral treatment, adults, tolerance, 3 nucleoside reverse transcriptase inhibitors, HIV infection, side effects, early treatment

## Abstract

**Introduction:**

Tenofovir (TDF) with emtricitabine (FTC) and zidovudine (ZDV) is a recognized alternate first-line antiretroviral (ART) regimen for patients who cannot start treatment with non-nucleoside reverse transcriptase inhibitors (NNRTIs). Clinical studies comparing TDF+FTC+ZDV to other regimens are lacking.

**Methods:**

Participants in a trial of early ART in Côte d'Ivoire (Temprano ANRS 12136) started treatment with TDF/FTC plus either efavirenz (EFV) or ZDV (HIV-1+2 dually infected patients and women refusing contraception or previously treated with nevirapine). We compared rates of upper digestive serious adverse events (sAEs) between TDF/FTC+EFV and TDF/FTC+ZDV patients during the first six months of treatment. sAEs were defined as either grade 3–4 AEs or persistent grade 1–2 AEs leading to drug discontinuation.

**Results:**

A total of 197 patients (76% women, median CD4 count 395/mm^3^) started therapy with TDF/FTC, 126 with EFV and 71 with ZDV. During the first six months of ART, 94 patients had digestive AEs (nausea/vomiting) of any grade (EFV 36/126, 29%; ZDV 58/71, 82%, p<0.0001), including 20 sAEs (EFV 3/126, 5%; ZDV 17/71, 24%, p<0.0001). In-patients on TDF/FTC+ZDV with digestive AEs, the median time to the first symptom was two days (IQR: 1–4). Plasma ZDV (Cmax) distributions and pill ZDV dosages were normal. Patients with digestive AEs had higher haemoglobin levels and tended to have higher body mass indices and more frequent past histories of cotrimoxazole (CTX) prophylaxis.

**Conclusions:**

We observed an unexpectedly high rate of digestive sAEs in West African adults, mostly women, who started a 3-nuc ART with TDF/FTC+ZDV in Côte d'Ivoire. These adults were participating in a trial of early ART and had much higher CD4 counts than those who currently routinely start ART in sub-Saharan Africa. They all received CTX concomitantly with ZDV. We suggest that further early prescriptions of TDF+XTC+ZDV should be carefully monitored and that whenever possible, the rate of early upper digestive adverse events should be compared to that occurring in-patients taking other drug regimens.

**Clinical Trial Number**: NCT00495651.

## Introduction

In sub-Saharan Africa, mortality in-patients on antiretroviral treatment (ART) remains high, especially within the year following treatment initiation, partly because most patients start ART when their CD4 count is well below 200 cells/mm^3^ [1–3]. In the near future, large programmes should promote earlier HIV diagnosis and optimize a linkage between HIV testing and access to care so that a larger percentage of patients can start treatment as soon as their CD4 count reaches 350 cells/mm^3^ [4–7]. In the longer term, people could be advised to start ART even earlier, should on-going trials of early ART demonstrate that there are more benefits than risks in doing so [8]. Several triple nucleoside reverse transcriptase inhibitor (3-NRTI) combinations have shown good efficacy and tolerance [6, 9]. Although less effective than non-nucleoside reverse transcriptase inhibitor (NNRTI)-based or protease inhibitor (PI)-based regimens in patients with high pre-ART plasma HIV RNA levels, 3-NRTI regimens have been considered acceptable first-line ART regimens for other patients [4–7]. In sub-Saharan Africa, 3-NRTI regimens could be especially attractive in patients who start ART early and who cannot receive NNRTIs. In settings where genotype testing is almost never available and where the number of drugs is limited, keeping PIs for potent second line treatment is crucial [4–7]. Because data on 3-NRTIs are limited, especially data comparing 3-NRTIs to other regimens, World Health Organization (WHO) experts called for more efficacy and tolerance data to support their use [4–7].

In this study, we compared the rates of serious adverse events (sAE) with tenofovir (TDF)/emtricitabine (FTC)+efavirenz (EFV) and tenofovir (TDF)/emtricitabine (FTC)+zidovudine (ZDV) given during the first six months of treatment in HIV-positive adults participating in a large trial of early ART in Abidjan, Côte d'Ivoire.

## Methods

### Temprano ANRS 12136

Temprano (http://clinicaltrials.gov/show/NCT00495651) is a multicentre, randomized, open-label trial to assess the benefits and risks of initiating ART earlier than currently recommended by the WHO, with or without a six-month isoniazid preventive therapy (IPT). The trial was launched in March 2008 in Abidjan and is still on-going. It will end in December 2014.

The trial inclusion criteria are as follows: HIV-1 or HIV-1+2 dual infected; age ≥18 years; signed informed consent; no on-going active tuberculosis; no on-going pregnancy; and no CD4 count or clinical stage-based criteria indicating the need to start ART immediately, according to WHO guidelines. The last criterion has evolved in line with WHO guideline updates: From March 2008 through November 2009, patients were enrolled if they had a CD4 count between 250 and 350 cells/mm^3^ and were at WHO clinical stage 1 or if they had a CD4 count between 351 and 600 cells/mm^3^ and were at WHO clinical stage 1, 2 or 3. Since December 2009, the clinical and immunological inclusion criteria are a CD4 count between 351 and 800 cells/mm^3^ and a WHO clinical stage 1, 2 or 3 [4–7].

Once enrolled, patients are randomized into four study arms: immediate ART, deferred ART, immediate ART plus six-month IPT, and deferred ART plus six-month IPT. Immediate ART means starting ART at Day 1, regardless of the CD4 count and clinical stage. Deferred ART means starting ART at any time during follow-up, as soon as WHO clinical and immunological criteria for starting ART are met [4–7]. IPT consists of a six-month course of isoniazide (300 mg once a day), starting at the Month-1 visit and culminating the Month-7 visit. The sample size required for this study was calculated to be 2072 participants. Each participant is followed for 30 months. The main outcome is the occurrence of a new episode of severe morbidity, including AIDS-defining diseases, non-AIDS-defining severe bacterial diseases, non-AIDS-defining cancers, and any events leading to death. The trial recruitment started in March 2008 and ended in July 2012. Each participant will be followed for at least 30 months. Written informed consent was received from all participants prior to inclusion. The trial received ethical approval from the Côte d'Ivoire National ethics committee.

### Patients

In the present study, we report baseline and follow-up characteristics up to the Month-6 visit for adults who enrolled in the Temprano trial from 1 March 2008 to 28 October 2008 and who started ART immediately with either TDF/FTC+EFV or TDF/FTC+ZDV. For each participant, the starting date of the study was the date of ART initiation. The closing date of the study was six months after ART initiation.

### Treatments

During the study period, patients randomized to start ART immediately were preferably given a fixed dose combination of tenofovir disoproxil fumarate 300 mg and emtricitabine 250 mg (Truvada^®^, Gilead, two tablets once a day) plus efavirenz 600 mg (Stocrin^®^, MSD, one tablet once a day). Patients with contra-indication to efavirenz (HIV-dual-positive patients, women who refused to take contraception and women who previously had received nevirapine monodose during a pregnancy) were given Truvada plus zidovudine 300 mg (one tablet twice a day, as provided by the Côte d'Ivoire national public pharmacy; during the study period, ZDV pills were purchased from three providers: Aurobindo, Ranbaxy and Cipla).

All patients with a CD4 count <500/mm^3^ were prescribed cotrimoxazole (CTX) prophylaxis (960/160 mg once a day) according to national guidelines.

### Visits and tests

At the end of the Day-0 visit, patients were given a one-month supply of pills and were asked to attend the scheduled trial visits on Day-8, Month-1, Month-2, Month-3 and every three months thereafter. CD4 count and plasma HIV-1 RNA were measured every six months. Patients had access to their study clinic at any time during the interval whenever they experienced clinical problems. Standardized questionnaires were used to record baseline and monthly characteristics. Transport, consultations, investigations, hospitalizations and drugs were free-of-charge.

Tests were performed as follows. First, at inclusion, all patients were assessed for serum transaminase, plasma HBs antigen and serum creatinine concentrations. Second, at inclusion and at Month-6, all patients were tested for blood cell count, CD4 cell count (True Count^®^ technique with a FACScan^®^, Becton Dickinson) and plasma HIV-1 RNA concentration (real-time PCR using Taq Man technology with an ABI Prism 7000, Applied Biosystems; detection limit 300 copies/ml). Third, during follow-up, all patients who experienced serious adverse events were tested for blood lactic acid and serum transaminase concentrations. Fourth, a subgroup of 25 patients was tested for ZDV drug plasma concentrations at the Day-8 visit. For this subgroup, a first sample to assess the minimum concentration (Cmin) was collected before dosage with 300 mg ZDV, and a second sample to assess the maximum concentration (Cmax) was collected one hour after administration of the drug. The Cmin and Cmax were estimated for ZDV, G-ZDV and TDF by high-performance liquid chromatography coupled with UV detection [10]. Finally, 15 tablets of ZDV were randomly tested for ZDV content, including five supplied by Aurobindo, five by Ranbaxy and five by Cipla [11].

### Definitions

All morbidity episodes, including adverse events, were referred for validation by an event documentation committee. Adverse events were graded according to the ANRS grading table [12]. For upper digestive adverse events, the criteria were as follows: (i) grade 1, transient nausea or vomiting (two or three episodes a day and duration of less than a week); (ii) grade 2, nausea leading to less than three days of inability to eat or vomiting (more than four episodes a day or duration more than a week); (iii) grade 3, nausea leading to more than three days of inability to eat or continuous vomiting for at least one day (requiring intravenous infusion of fluids); (iv) grade 4, nausea leading to permanent inability to eat and requiring hospitalization or severe vomiting inducing hypovolemia. We further classified occurrences of either nausea or vomiting as significant adverse events (grade 3–4), or persistent (grade 1–2, leading to treatment discontinuation) and moderate events (grade 1–2, without treatment discontinuation).

### Statistical analysis

Patients baseline characteristics, cumulative rates of adverse events within first month of treatment, drug intake characteristics within first month (missed pill, treatment interruption), and patients status at Month-6 (vital status, CD4 change since baseline, percentage with undetectable plasma viral load) were compared between treatment regimens (TDF/FTC and ZDV vs. TDF/FTC and EFV) using Fisher exact, Chi-square, Wilcoxon sum rank test or Mantel Haenszel trend test when appropriate. Baseline characteristics, pills characteristics (manufacturer, pill dosages) and plasma drug concentrations (the latter, in a sample of patients) were compared between patients who experienced adverse events and those who did not among patients who received TDF/FTC and ZDV. Tests were two-sided, with a statistical significance limit of 5%. Analyses were performed with SAS software (version 9.1, SAS Institute).

## Results

### Baseline characteristics and follow-up

From March to September 2008, 400 patients were enrolled in the Temprano trial, 199 of whom were randomized to start ART immediately. Two patients were excluded from the present analysis because they started ART with TDF/FTC+lopinavir/ritonavir. The remaining 197 patients (women: 150, men: 47) were included in the analysis, of whom 71 (women: 69, men: 2) started TDF/FTC+ZDV and 126 (women: 81, men: 45) started TDF/FTC+EFV. Compared to patients on EFV, those on ZDV were more frequently female and at WHO clinical stage 1. They were also younger and had lower pre-ART plasma HIV-1 RNA concentrations and higher pre-ART body mass indexes. At the end of the first six months of follow-up, 98% of patients were alive and in active follow-up (Table 1).

**Table 1:** Patient baseline and follow-up characteristics (n=197)

	TDF/FTC +ZDV (n=71)	TDF/FTC +EFV (n=126)	*p*
*Baseline characteristics*			
Female, n (%)	69 (97%)	81 (64%)	<0.001
Age (years), median (IQR)	32 (29–37)	39 (31–45)	0.02
WHO clinical stage, n (%)			0.003
Stage 1	59 (83%)	81 (64%)	
Stage 2	12 (17%)	35 (28%)	
Stage 3	–	10 (08%)	
Body mass index (kg/m^2^), median (IQR)	24.0 (21.1–26.1)	22.3 (20.2–25.2)	0.04
CD4 count (cells/mm^3^), median (IQR)	388 (299–446)	395 (323–498)	0.09
Plasma HIV-1 RNA (log_10_ copies/ml), median (IQR)	4.6 (3.9–5.1)	4.9 (4.3–5.4)	0.007
HIV subtype, n (%)			0.10
HIV 1 only	66 (93%)	124 (98%)	
HIV 1–2 dual	5 (7%)	2 (2%)	
Positive serum HBs antigen, n (%)	9 (13%)	15 (12%)	0.87
Haemoglobin (g/l), median (IQR)	106 (98–113)	108 (99–120)	0.19
Creatinine clearance<60 ml/min	0	4 (3%)	0.30
Serum transaminases>1.25×ULN, n (%)	1 (1%)	5 (4%)	0.42
Cotrimoxazole started before inclusion, n (%)	41 (58%)	79 (63%)	0.49
Time on CTX (months), median (IQR)	9.4 (2.2–27.5)	7.3 (0.8–21.9)	0.26
Positive QuantiFERON^®^-TB Gold test, n (%)	14 (20%)	32 (28%)	0.25
*Status at Month-6*			
Dead	0	2 (2%)	
Alive and in active follow-up	67 (94%)	121 (96%)	0.28
Lost to follow-up	4 (6%)	3 (2%)	

n: number; %: percentage; IQR: interquartile range; ULN: upper limit of normal; ml/min: millilitres per minute; g/l: grams per litre; kg/m^2^: kilograms per square metre; mm^3^: cubic millimetre: HBs antigen: hepatitis B surface antigen.

TDF: tenofovir; FTC: emtricitabine; ZDV: zidovudine; EFV: efavirenz.

Women were younger than men (mean age 34 years in women, 41 years in men; p<0.0001). There were no significant differences between genders in the distribution of baseline body mass index (p=0.50), CD4 count (p=0.66), plasma viral load (p=0.05) and WHO clinical stage (p=0.69).

### Adverse events

Overall, 94 digestive adverse events (AEs) were recorded, including 38 nausea events without vomiting (40%) and 56 with vomiting (60%). The median time to digestive AE after ART initiation was two days (interquartile range [IQR]: 1; 4) in patients on ZDV and four days (IQR: 1; 19) in patients on EFV (p=0.01). Of the 94 digestive AEs, 74 (79%) were classified as moderate AEs, and 20 (21%) as serious AEs (sAEs). Of the 20 sAEs, 15 (ZDV: 12, EFV: 3) were grade 3 or 4 AEs, and five (all ZDV) were prolonged grade 2 AEs leading to permanent discontinuation of the drug. Patients on ZDV had significantly more frequent digestive AEs of any grade (82% vs. 28%, p<0.001) and more frequent digestive sAEs (24% vs. 2%, p<0.001) than patients on EFV (Table 2).

**Table 2:** Early upper gastrointestinal adverse events, by drug regimen (n=197)

	TDF/FTC +ZDV (n=71)‡	TDF/FTC +EFV (n=126)‡‡	*p*
Adverse events within first month of treatment, n (%)			0.0001
None	13 (18%)	90 (71%)	
Moderate*	41 (58%)	33 (26%)	
Serious**	17 (24%)	3 (2%)	
Grade 3 or 4†	12 (17%)	3 (2%)	
Grade 2>1 month & drug discontinuation, n (%)	5 (7%)	0	
Characteristics recorded at Month-1 visit, n (%)			
Treatment interruption ≥7 days within first month	3 (4%)	1 (0.8%)	–
At least 1 pill missed (last 4 days questionnaire)	14 (21%)	10 (8%)	0.02
Characteristics recorded at Month-6 visit			
CD4 change at Month-6 (cells/mm^3^), median (IQR)	+90 (−8; +175)	+123 (−12; +226)	0.28
HIV-1 RNA>300 copies/ml at Month-6, n (%)	14 (21%)	15 (12%)	0.13

n: number; %: percentage; IQR: interquartile range; mm^3^: cubic millimetre.

TDF: tenofovir; FTC: emtricitabine; ZDV: zidovudine; EFV: efavirenz.

* Moderate: grade 1 or 2, without drug discontinuation.

** Serious: grade 3 or 4, or persistent grade 2>1 months leading to drug discontinuation.

† Grade 3–4 vomiting, n=10; grade 3–4 nausea with grade 1–2 vomiting, n=5.

‡ 69 out the 71 patients (97%) on TDF/FTC+ZDV were women. Of the two men who started on TDF/FTC+ZDV, one had moderate digestive AEs and continued the treatment, and the other one had grade 2>1 month AEs leading to drug discontinuation.

‡‡ 81 out the 126 patients (64%) on TDF/FTC+EFV were women. Among the 81 women who started on TDF/FTC+EFV, 27 (33%) had moderate digestive AEs and 3 (4%) had serious digestive AEs.

An additional nine non-digestive sAEs were recorded, including four in-patients on ZDV (anaemia, n=3; non-obstructive cardiomyopathy, n=1) and five in patients on EFV (dizziness, nightmare or acute delirium, n=3; renal insufficiency or nephrotic syndrome, n=2) (p=0.70).

### Gender issue

Among the 150 participating women, there were 86 digestive AEs, including 66 (77%) classified as moderate AEs and 20 (23%) as serious AEs (sAEs). Of the latter, 15 (ZDV: 12, EFV: 3) were grade 3 or 4 AEs, and 5 (all ZDV) were prolonged grade 2 AEs leading to permanent discontinuation of the drug. Women on ZDV had significantly more frequent digestive AEs of any grade (81% vs. 37%, p<0.001) and more frequent digestive sAEs (25% vs. 4%, p<0.001) than women on EFV.

### Characteristics associated with digestive AEs in patients on ZDV

For digestive AEs, no patients had abnormal blood lactic acid or abnormal serum liver enzyme levels at the onset of the event. Among patients who started ZDV, those who experienced digestive AE had significantly higher baseline haemoglobin values; they also tended to have started cotrimoxazole more frequently prior to inclusion in the study and higher BMIs than those who did not experience digestive AEs. There was no association between digestive AE occurrence and other variables, including ZDV pill providers (Table 3).

**Table 3:** Characteristics of patients with and without early upper gastrointestinal adverse events on TDF-FTC-ZDV (n=71)

	Upper gastrointestinal adverse event within first month of treatment		
		
	No (n=13)	Yes (n=58)	*P*
Female, n (%)	13 (100%)	56 (97%)	1.00
Age (years), median (IQR)	31 (29–34)	32 (29–37)	0.78
WHO clinical stage, n (%)			0.44
Stage 1	12 (92%)	47 (81%)	
Stage 2 or 3	1 (8%)	11 (19%)	
Body mass index (kg/m^2^), median (IQR)	22.5 (21.4–24.2)	24.4 (21.1–26.4)	0.13
CD4 count (cells/mm^3^), median (IQR)	426 (357–444)	378 (291–446)	0.27
HIV serotype, n (%)			0.57
HIV-1 only	13 (100%)	53 (91%)	
Dual	–	5 (9%)	
Cotrimoxazole started before ART, n (%)	4 (31%)	37 (64%)	0.06
Time on Cotrimoxazole (months), median (IQR)	7.0 (0.3–20.6)	9.4 (2.4–36.7)	0.38
Positive serum HBs antigen, n (%)	–	9 (16%)	0.19
Haemoglobin (g/l), median (IQR)	98 (90–102)	108 (101–115)	0.01
Creatinine clearance (ml/min)			0.99
<60	1 (8%)	6 (11%)	
≥60	12 (92%)	51 (89%)	
Serum transaminases >1.25×ULN, n (%)	–	1 (2%)	0.99
Zidovudine pill manufacturer, n (%)			0.79
ZDV 300 mg Ranbaxy or Aurobindo	5	24	
ZDV 300 mg Cipla	6	21	
ZDV 250 mg GSK	2	13	

ULN: upper limit of normal; ZDV: zidovudine; CTX: cotrimoxazole;

n: number; %: percentage; IQR: interquartile range; ULN: upper limit of normal; ml/min: millilitres per minute; g/l: grams per litre; kg/m^2^: kilos per square metre; mm^3^: cubic millimetre: HBs antigen: hepatitis B surface antigen.

Of the 15 patients with prolonged grade 1–2 digestive AE, one permanently discontinued ZDV without prior interruption of CTX, and 14 patients interrupted CTX prophylaxis during two weeks prior to the decision of stopping ZDV. Among these 15 patients, the digestive symptoms resolved in 11 (79%) and persisted in four. In the latter four patients, symptoms resolved after ZDV discontinuation.

### Pill ZDV concentrations and blood measurements

The mean ZDV concentration was 340 mg (range: 321–404) in tablets manufactured by Aurobindo, 324 mg (range: 314–346) in tablets manufactured by Ranbaxy and 298 mg (range: 281–316) in tablets manufactured by Cipla, with no significant differences between providers.

In the 21 patients with digestive AE who had plasma drug concentration measurements, ZDV Cmax, G-ZDV Cmax, tenofovir Cmin and tenofovir Cmax appeared to be in the range of normal values (Table 4).

**Table 4:** Plasma concentration of antiretroviral drugs in a sample of patients who started TDF-FTC+ZDV (n=25)

	Upper gastrointestinal adverse event within first month of treatment		
		
	No (n=4)	Yes (n=21)	*p*
ZDV Cmax (ng/ml), median (IQR)	1294 (735–1870)	1184 (831–1486)	0.50
G-ZDV Cmax (ng/ml), median (IQR)	9258 (6787–13604)	7054 (5071–8956)	0.24
Tenofovir, median (IQR)			
Cmin (ng/ml)	48 (28–77)	64 (26–88)	0.74
Cmax (ng/ml)	39 (29–59)	60 (29–85)	0.48

ZDV: zidovudine; G-ZDV: Glucuronide metabolite of ZDV; Cmin (minimum concentration): Measured before pill intake; Cmax (maximum concentration): Measured 1 hour after ZDV pill intake; IQR: interquartile range; ng/ml: nanograms per millimetre.

### Six-month outcomes

**Figure 1:**
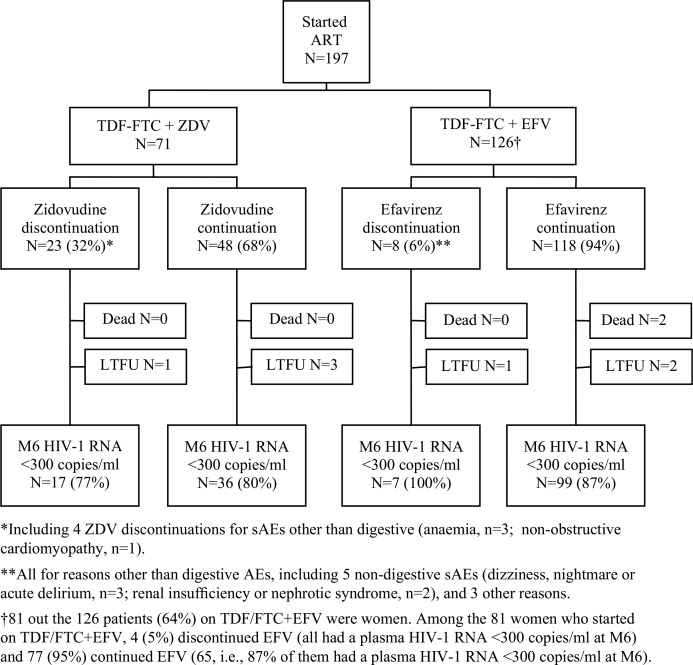
Early drug discontinuation and viral load suppression at six months in patients who started ART with TDF-FTC-EFV or TDF-FTC-ZDV. LTFU: lost-to-follow-up; M6: Month-6.

At the Month-1 visit, a higher percentage of patients on ZDV reported having interrupted their treatment for more than seven days and/or having missed at least one pill during the past four days (Table 2). At the Month-6 visit, there was a trend towards a lower gain in CD4 and a higher percentage of patients with detectable viral load in patients on ZDV compared to those on EFV (Table 2). Overall, 32% patients on ZDV and 6% patients on EFV switched to another regimen during the first six months (Figure 1).

## Discussion

According to current knowledge, there are two acceptable 3-NRTI-based regimens: TDF-FTC or 3TC (XTC)-ZDV and ABC-XTC-ZDV. Compared to the latter, the former is cheaper and is considered to be better tolerated and does not need HLA-B5701 screening prior to prescription [13, 14].

In this study, we observed an unexpectedly high rate of upper digestive adverse events in patients, mostly women, who started treatment with TDF/FTC+ZDV. These adverse events occurred during the very first days of treatment. Some of them were explosive, making any drug intake impossible and leading patients to immediately stop taking the drug. Others were less severe and did not preclude patients from continuing the drug; however, these AEs did not resolve over time. Because the lingering nausea or vomiting could lead to poor patient compliance over the long term, these patients were switched to a better-tolerated regimen. Overall, these grade 3–4 or persistent grade 1–2 adverse events were significantly more frequent in patients on TDF/FTC+ZDV compared to those on TDF/FTC+EFV. After carefully reviewing the evidence, the Temprano trial data safety monitoring board recommended that TDF/FTC+ZDV should no longer be given as first-line treatment to new participants in the trial. From December 2008 onwards, all new patients included in the Temprano trial who could not receive EFV as part of their first line treatment were prescribed LPV/r.

We found normal blood concentrations of ZDV, G-ZDV and TDF [11, 15] in a sample of patients with digestive adverse events and normal content of ZDV in pills sampled at random from the stock dedicated to the patients included in the trial. We found no evidence that the digestive adverse events were associated with any particular pill manufacturer.

Our trial was not designed to compare TDF/FTC+EFV with TDF/FTC+ZDV, especially as TDF/FTC+ZDV was given to patients for whom treatment with EFV was contraindicated. Furthermore, our sample size was limited because TDF/FTC+ZDV treatments were stopped prematurely, thus limiting any adjusted comparison between regimens. As a consequence, we could not deeply explore the reasons for the high rate of sAE occurrence. Nonetheless, we could make two observations that led to several hypotheses.

First, in our study, patients on TDF/FTC+ZDV who experienced digestive AEs had higher haemoglobin levels and tended to have higher BMIs than patients on TDF/FTC+ZDV who did not. Because they were participating in an early ART trial, our patients started ART at much higher CD4 counts than most adults who have started ART in sub-Saharan Africa so far. In the DART trial, held in Uganda and Zimbabwe, 75% of participants were at WHO stage 3 or 4, and the mean pre-ART CD4 count was 101 cells/µl. In this trial, no digestive sAEs were reported from patients taking ZDV/3TC+TDF [16]. Thus, our first hypothesis is that digestive AEs may be more frequent when TDF+XTC+ZDV treatment is started early. We found two pieces of evidence consistent with this hypothesis. First, in a pilot study of 24 HIV-positive patients in France who started TDF+3TC+ZDV with a mean CD4 count of 443 cells/µl, serious digestive AEs led to treatment interruption or switch in three patients [7]. Second, high rates of upper digestive events were also reported from post-exposure studies in which HIV negative patients received ZDV-containing ART to prevent HIV transmission [17, 18]. Of note, in these studies, women had a higher risk of nausea than men [17, 18]. In our study, 97% of patients who took TDF/FTC+ZDV were women.

Second, in our study, patients who had started CTX before inclusion tend to have more frequent sAEs, and digestive sAEs resolved in a majority of patients with grade 1–2 sAE who stopped CTX. Thus, competitive metabolism of ZDV with CTX may be occurring. CTX undergoes glucuronidation, which is also the predominant pathway for metabolizing ZDV [15, 19]. A small study in the United States reported a trend towards higher rates of ZDV adverse events in African American patients than in other patients [20], possibly due to interethnic polymorphism in the major enzyme of glucuronidation, UDP-glucuronosyltransferase [21, 22]. However, pharmacokinetic and clinical studies suggest that the effect of CTX on ZDV glucuronidation is weak and has no clinical consequences in the absence of liver impairment [23–28]. Furthermore, we did not find such a high rate of digestive AEs in previous studies in which CTX was systematically given in combination with ZDV in Côte d'Ivoire [29]. If the interaction between CTX and ZDV did have a role to play in the high rate of digestive AEs that we observed here, other co-factors must have acted as catalysts.

## Conclusions

We observed an unexpectedly high rate of digestive sAEs in West African adults, mostly women, who started a 3-nuc ART with TDF/FTC+ZDV in Côte d'Ivoire. These adults were participating in a trial of early ART and had much higher CD4 counts than those who currently routinely start ART in sub-Saharan Africa. They all received CTX concomitantly with ZDV.

Large studies on patients on TDF+XTC+ZDV, as well as comparisons between TDF+XTC+ZDV and other ART regimens, have been rare so far, especially in sub-Saharan Africa. Most of the previous existing reports have been from patients with low pre-ART CD4 counts.

We suggest that further early prescriptions of TDF+XTC+ZDV should be carefully monitored and that whenever possible, the rate of early upper digestive adverse events should be compared to that occurring in patients taking other drug regimens. Such comparison should be stratified based on gender in order to clarify whether the risk of adverse events might be higher in women compared to men.
